# Thiamine deficiency in pregnancy and lactation: implications and present perspectives

**DOI:** 10.3389/fnut.2023.1080611

**Published:** 2023-04-20

**Authors:** Ozaifa Kareem, Sobia Nisar, Masood Tanvir, Umar Muzaffer, G. N. Bader

**Affiliations:** ^1^Department of Pharmaceutical Sciences, University of Kashmir, Srinagar, India; ^2^Department of Medicine, Government Medical College, Srinagar, India

**Keywords:** thiamine deficiency, pregnancy, infantile beriberi, thiamine, thiamine diphosphate

## Abstract

During pregnancy, many physiologic changes occur in order to accommodate fetal growth. These changes require an increase in many of the nutritional needs to prevent long-term consequences for both mother and the offspring. One of the main vitamins that are needed throughout the pregnancy is thiamine (vitamin B1) which is a water-soluble vitamin that plays an important role in many metabolic and physiologic processes in the human body. Thiamine deficiency during pregnancy can cause can have many cardiac, neurologic, and psychological effects on the mother. It can also dispose the fetus to gastrointestinal, pulmonological, cardiac, and neurologic conditions. This paper reviews the recently published literature about thiamine and its physiologic roles, thiamine deficiency in pregnancy, its prevalence, its impact on infants and subsequent consequences in them. This review also highlights the knowledge gaps within these topics.

## 1. Introduction

Pregnancy is a dynamic, anabolic state characterized by a series of small, continuous physiologic changes that affect the metabolism of all nutrients (1). Pregnancy brings about a slew of anatomical and physiologic changes that have an impact on the body’s metabolism (2). These changes in the mother’s body occur to accommodate and promote intrauterine fetal growth and development (3). The metabolic physiology during pregnancy changes dramatically to provide adequate blood, nutrition and oxygen to the growing fetus and prepare the mother’s body for childbirth and lactation (4). To maintain maternal metabolism and tissue growth, nutritional needs rise throughout pregnancy (5). Poor maternal nutrition during pregnancy has several long-term negative repercussions on the health of the mother and her offspring, including higher neonatal morbidity and mortality (6). To promote necessary one-carbon metabolism for DNA methylation processes during embryogenesis and normal fetal development, sufficient dietary quantities of micronutrients like folate, riboflavin, thiamine, vitamin B6, and vitamin B12 are required (7). Thus, pregnant women are prone to secondary nutritional deficiency due to higher metabolic demand.

Thiamine, also known as aneurin or vitamin B1, is a water-soluble vitamin and a vital micronutrient for human beings (8). It plays an essential role in energy metabolism especially in Kreb’s cycle (9). Thiamine is not synthesized endogenously in humans and its supply is entirely dependent upon dietary intake, though some intestinal bacteria are known to produce minute amounts of the vitamin (10). The half-life of thiamine is short (1–12 h) and the body stores of thiamine deplete fast (within 2 weeks of deprivation), thus a regular ample supply is mandatory to maintain the tissue thiamine levels (11). Whole grains, yeasts, meat, legumes, and nuts are the best sources of thiamine (8). Thiamine deficiency is predominant in populations where the diet chiefly comprises of low thiamine sources such as milled white cereals, polished rice (the thiamine-rich husk is removed by polishing), and wheat flour, and where other rich thiamine sources are infrequently consumed (12). Thiamine requirement increases during pregnancy due to the sequestration of the vitamin by the fetus and placenta (13).

Thiamine deficiency can cause neurological and cardiovascular complications which have the potential to be serious, and sometimes fatal. The group of disorders caused by thiamine deficiency are collectively known as beriberi (14). A mild deficiency of thiamine can sometimes cause subtle symptoms that are easily disregarded. However, thiamine in pregnant and postpartum women can present as polyneuropathy (15) or a more fatal form of infantile beriberi (16). Infants are especially vulnerable to the effects of thiamine deficiency during the first few months of life, and exclusively breastfed infants of thiamine-deficient mothers are at the highest risk (17). During pregnancy, dependence on a high carbohydrate diet, poor dietary sources of thiamine like polished white rice, cassava and a monotonous diet leads to a thiamine-deficient state in women (18, 19). Further, during lactation, dietary taboos, postpartum restrictive diet and consumption of thiaminase-containing foods like fermented fish, tea leaves and betel nut result in a life-threatening form of beriberi in infants (20, 21). Infantile beriberi is the most severe, unfavorable effect of low or marginal thiamine levels in mothers wherein the clinical signs appear more quickly in newborns than in adults due to the accelerated growth and development occurring during this stage of life (22). Thiamine deficiency in women of reproductive age, pregnant and lactating mothers has been reported from low- and middle-income countries (LMIC) (23, 24), especially in under-resourced environments and food-scarce situations like war, famine, political embargos etc. (25, 26). Thiamine deficiency during pregnancy and lactation has been reported in Southeast Asia (27–29) and sub-Saharan Africa (30). Infantile beriberi has also been recognized in several regions of central and Southeast Asia, especially in Myanmar, Laos, and Cambodia (26, 31–34). The regions where both clinical and subclinical thiamine deficiencies have been reported or are likely to be ubiquitous, interventions in terms of food fortification, supplementation, diet optimization, health education and behavioral changes are strategic policies that need to be implemented to overcome the burden of thiamine deficiency disorders.

This review summarizes the present knowledge on thiamine deficiency in pregnant/lactating women and infants and the importance of evaluating thiamine deficiency early on to overcome its adverse implications in infants and children. The review is also aimed at addressing the gap in understanding the development of thiamine deficiency during pregnancy and lactation and the potential dietary and physiological changes in these states that contribute to pathological manifestations of this deficiency in mothers as well as infants. Besides, this narrative review explores some important queries like (1) Physiological changes during pregnancy and lactation contributing to the development of thiamine deficiency (2) Prevalence of thiamine deficiency in women and infants worldwide (3) Genetic factors that contribute to the development of thiamine deficiency (4) Clinical consequences of thiamine deficiency in pregnancy/lactation as well as among infants (5) Strategies to be adopted for boosting thiamine consumption in areas where clinical and subclinical deficits are common.

## 2. Methodology

The study populations of interest included pregnant and lactating women with thiamine deficiency and/or infants with thiamine deficiency. The neonatal complications and genetic derangements associated with thiamine transport genes were also included in this review. The animal studies and populations other than pregnant/lactating/infants were excluded from the review. To acquire information about thiamine deficiency in the population of interest, an electronic worldwide literature search was conducted. Pubmed, Google Scholar, Scopus, and Google were among the databases used. Search terms or keywords included “thiamine deficiency,” “beriberi,” “thiamine deficiency pregnancy,” “thiamine deficiency lactating,” “infantile beriberi,” “wet beriberi,” “dry beriberi,” “prevalence thiamine deficiency,” “thiamine transporters and thiamine deficiency.” The literature search yielded 373 results without language or geographic restrictions out of which 294 meaningful publications were included in the study according to the aim of this review.

## 3. Physiological role of thiamine in humans

Thiamine is an essential, hydrophilic, sulfur-containing vitamin that belongs to the vitamin B complex family (35). Thiamine is rapidly absorbed from food but in an alkaline environment with a pH > 8 or at high temperatures, thiamine is readily eliminated. Moreover, the presence of anti-thiamine factors in a variety of foods like raw fish, ferns, African silkworm larvae, and beverages prevents the absorption of thiamine by catalyzing its cleavage, thus vitiating its activity (36, 37). However, these thiaminases are heat sensitive and are rendered inactive by cooking processes. Polyhydroxyphenols are another anti-thiamine component that inactivates thiamine through an oxyreductive mechanism. Unlike thiaminases, these polyhydroxyphenols, which include caffeic and tannic acids present in coffee, tea, betel nuts, black currents, red cabbage, blueberries, and Brussels sprouts are heat stable. These compounds can be destroyed by the presence of divalent cations, like calcium and magnesium, while reducing compounds, such as vitamin C, can inhibit its degradation (38–40).

Thiamine occurs in the body as free thiamine and in numerous forms, including thiamine monophosphate (ThMP), thiamine diphosphate (ThDP), and thiamine triphosphate (ThTP) (41–43). ThDP, commonly known as thiamine pyrophosphate (TPP), is an active metabolite accounting for around 80% of total thiamine in the body. ThDP serves as an essential cofactor in several enzyme complexes involved in energy metabolism especially carbohydrates and amino acids (44). These enzyme complexes include the pyruvate dehydrogenase complex (which converts pyruvate to acetyl-CoA), the -ketoglutarate dehydrogenase complex (which converts -ketoglutarate to succinyl-CoA), and the branched-chain -keto acid dehydrogenase complex (which converts branched-chain -keto acids to the corresponding acyl-CoAs), the -pentose phosphate pathway (cytosolic transketolase), and in α-oxidation of phytanic acid (2-hydroxy acyl-CoA lyase) (35). In addition, several other cellular activities, such as the generation of nucleic acid precursors, myelin, and neurotransmitters (e.g., acetylcholine, glutamate and gamma-aminobutyric acid), as well as antioxidant defense, require thiamine for TPP-dependent transketolase (45). Essentially, the deficiency of thiamine limits the supply of enzymes to the Krebs cycle leading to decreased adenosine triphosphate (ATP) synthesis, oxidative damage, and cell death (46).

### 3.1. Role of TPP-dependent enzymes

#### 3.1.1. Cytosol

In the cytosol, TPP acts as a cofactor for transketolase (TKT), a crucial enzyme of the non-oxidative branch of the pentose phosphate pathway (PPP) (47). This pathway utilizes glucose and produces ribose-5-phosphate and nicotinamide adenine dinucleotide phosphate (NADPH) (48). The vital role of ribose-5-phosphate in DNA and RNA production emphasizes the need for thiamine in high-proliferating tissues. Thus, improper availability or utilization of thiamine will lead to oxidative stress, decreased cell proliferation, and reduced fatty acid synthesis (especially myelin) which can have severe effects during brain development (49).

#### 3.1.2. Peroxisomes

The α-oxidation of branched fatty acids is carried out by TPP-dependent enzyme 2-hydroxy acyl-CoA lyase 1 (HACL1) along with Mg^2+^ (50, 51). HACL1 serves to break down the phytanic acid, by an initial α-oxidation, to 2-methyl-branched pristanic acid (2,6,10,14-tetramethylpentadecanoic acid) and formyl CoA. These components can get oxidized to produce CoA derivatives that can enter the citric acid cycle and CO2 respectively (52). The impairment of the breakdown of phytanic acid, due to deficiency of TPP, results in triglyceride accumulation and is pathognomonic for Refsum’s disease (53). This leads to symptoms, such as peripheral polyneuropathy, visual disturbances, cerebellar ataxia, and sometimes, epiphyseal dysplasia and cardiac dysfunction which closely mimic the symptomatology of beriberi (54).

#### 3.1.3. Mitochondria

In mitochondria, TPP is a crucial cofactor for three different 2-oxoacid dehydrogenase complexes, viz, pyruvate dehydrogenase, (PDH) α-ketoglutarate dehydrogenase (αKDGDH), and branched-chain α-ketoacid dehydrogenase (BCKDH) that despite utilizing diverse metabolic pathways, have similar macromolecular assembly structures and catalyze similar decarboxylation processes (55).

#### 3.1.4. Pyruvate dehydrogenase complex

A critical step in carbohydrate metabolism involves the TPP-dependent oxidative decarboxylation of pyruvate to acetyl-CoA, CO2, and NADH by the PDH enzyme complex, which then enters the Krebs cycle (56). This step is heavily reliant on thiamine, the deficiency of which leads to the failure of pyruvate to enter the TCA cycle, driving the system into the oxidation of glucose to pyruvate, which is finally diverted to the production of lactate and lactic acid by the lactate dehydrogenase enzyme (57, 58). This can present as acute life-threatening metabolic acidosis, peripheral and central neuropathies, seizures and intellectual disability (59–61).

#### 3.1.5. α-ketoglutarate dehydrogenase complex

The TPP-dependent enzyme complex catalyzes the formation of succinyl-CoA and reduces nicotinamide adenine dinucleotide (NADH) (62). During low TPP levels, KDDH activity is reduced, decreasing energy production, accumulating glutamate, and obstructing oxidative metabolism, resulting in neurodegeneration (63, 64).

#### 3.1.6. Branched-chain α-keto dehydrogenase complex

The metabolism of three nutritionally essential branched-chain amino acids, valine, leucine, and isoleucine, is dependent upon the TPP-dependent BCKDH enzyme (65). These amino acids are utilized in protein synthesis and provide nitrogen for *de novo* glutamate synthesis (66). The inadequate supply of thiamine impairs the metabolism of these branched-chain amino acids resulting in the accumulation of branched-chain keto acids (67). This in turn leads to the increased β-oxidation of fatty acids and hence metabolic dysfunction including dyslipidemia (68).

### 3.2. Transporters

Thiamine, like most hydrophilic micronutrients, is absorbed most effectively in the mucosal cells of the upper jejunum and to a lesser extent from the duodenum and ileum (69). Additionally, dietary proteins are digested in the digestive tract, releasing thiamine (70). Thiamine absorption occurs by a dual mechanism that is saturable at low physiological concentrations (oral intake <5 mg) and diffusive at higher concentrations (39). The human intestinal absorption of thiamine is regulated by the two high-affinity carriers: human thiamine transporters-1 (hTHTR-1), encoded by Solute Carrier Family 19 Member 2 (SLC19A2), and hTHTR-2, encoded by SLC19A3 (71–73). SLC19A2 mutations cause thiamine-responsive megaloblastic anemia (TRMA), which is accompanied by hyperglycemia and sensorineural deafness (74). The hTHTR-1 facilitates the transport of thiamine from the intestine to the portal circulation via the basolateral membrane and thus enters erythrocytes (42, 75). These cells contain about 80% of thiamine in the form of TPP while very low concentrations are present in plasma (76). Thiamine concentrations are highest in the heart (0.28–0.79 mg/100 g), followed by kidneys (0.24–0.58 mg/100 g), liver (0.20–0.76 mg/100 g), and brain (0.14–0.44 mg/100 g), with brain concentrations lasting the longest (36). The half-life of thiamine is only 9.5–18.5 days while the human body does not retain thiamine at concentrations greater than 30 mg (37, 42). The low reservoir of thiamine, combined with its short half-life and continuous demand for utilization in metabolic activities, necessitates steady thiamine consumption (77). With marginal intake, the symptoms of thiamine deficiency may appear within 72 h (78). The reuptake of thiamine from urine is facilitated by hTHTR1 and hTHTR2 in the basolateral membrane and brush border membrane and renal tubular cells. Long-term diuretic use has been linked to thiamine deficiency. Loop diuretics, such as furosemide, cause large thiamine loss, up to 2 times the baseline (79). Thiamine is also eliminated through feces, sweat and breast milk (41, 75, 80). Thiamine levels in breast milk are normally in the range of 0.14–0.21 mg/L, although they vary depending on the diet (81).

### 3.3. Biomarkers/analysis

There are two principal methods of evaluating the thiamine status: assessment of the degree of ThDP saturation of a thiamine-dependent enzyme [erythrocyte transketolase (ETK) assay] and evaluation of the metabolites of thiamine inaccessible tissues (81). The erythrocyte transketolase (ETK) assay is the widely used, most comprehensive method of determining thiamine status as it reveals the functionality of the vitamin (23). Patients with an increase in ETKA of more than 25% are at high risk of TD, those with increases of 16–25% are at moderate risk of TD, and those with activity coefficients of less than 15% have adequate thiamine status (37, 82). Direct assessment of ThDP in whole blood by High-Performance Liquid Chromatography (HPLC) is the method of choice with high precision, sensitivity and specificity (83, 84). Since 80% of the total thiamine content of whole blood is present as active ThDP in red blood cells making this method superior and more reflective of body stores of thiamine. For normal healthy individuals, the expected concentration of ThDP in whole blood is 70-180 nmol/L (82, 85). The metabolites of thiamine can also be determined by HPLC embedded with ultraviolet-visible spectroscopy (UV/VIS) or fluorescence detectors and Liquid chromatography-mass spectrometry (LC-MS) (86).

### 3.4. Recommended intake

Since the human body does not store thiamine for long, an adequate daily supply is required to maintain homeostasis. The recommended nutrient intake (RNI) is the daily intake, which meets the nutrient requirements of almost all healthy individuals in an age- and sex-specific population group (87). RNI of thiamine is 1.2 mg/day and 1.1 mg/day for men and women respectively. During pregnancy and lactation, the requirement increases to 1.4 and 1.5 mg/day respectively. The intake for infants is set at 0.2 mg/day (0–6 months) and 0.3 mg/day (7–12 months) (81). In addition to this, the thiamine requirement is increased after the consumption of a high carbohydrate diet; for every 2,000 kcal consumed daily, the minimum thiamine requirement is set at 0.66 mg (88).

## 4. Physiology of pregnancy and its impact on thiamine

Pregnancy (also known as gestation or gravidity) is the time between conception and birth during which an embryo and, eventually, a fetus develops inside a woman’s uterus. During this period, maternal physiology changes dramatically affecting all systems of the body, which are often interrelated (3). These physiological changes are designed to provide adequate oxygen and nutrients to both the mother and the developing fetus (89). In order to fulfill the growing maternal, fetal and placental demands, pregnancy puts a unique strain on micronutrient absorption and dissemination. It has been well established that improved nutrition during pregnancy and early postnatal life is critical for general wellbeing and minimizes the risk of chronic diseases later in life (90, 91).

During pregnancy, a woman undergoes numerous physiological adaptations chiefly in the hematological, renal, cardiovascular, respiratory, digestive, and endocrine systems (92). One of the most significant changes during pregnancy includes the expansion of body fluid volume. A total of 6.5–8.5 L increment in body fluid volume occurs, most of which occur by 34 weeks (93). This increase in plasma volume is much higher than the consequent rise in red blood cell mass, leading to hemodilution and a fall in hemoglobin concentration, hematocrit, and red blood cell count (94, 95). The concentration of all clotting factors, except XI and XIII, is increased during pregnancy with a corresponding decrease in endogenous anticoagulants such as antithrombin and protein S, leading to a physiological hypercoagulable state (96). By 8 weeks of pregnancy, the cardiac output increases by 20% primarily by vasodilatation due to endothelium-dependent factors, nitric oxide synthesis, and vasodilatory prostaglandins (PGI_2_) (97). Renal plasma flow and glomerular filtration rate (GFR) both rise by 40–65% and 50–85%, respectively, as a result of renal vasodilation during pregnancy. Additionally, an increase in plasma volume produces a decrease in oncotic pressure in the glomeruli, resulting in a rise in GFR (98). Furthermore, arterial underfilling leads to activation of the renin-angiotensin-aldosterone system resulting in sodium and water retention in the kidneys. This creates a hypervolaemic, hypoosmolar state of pregnancy (99). Also, nausea and vomiting are relatively frequent pregnancy-related complaints, affecting nearly 50–90% of pregnancies (100). All these changes, in association with hyperemesis gravidarum (a severe form of pregnancy-related nausea and vomiting), lead to > 5% weight loss, dehydration, ketonuria, nutritional deficiencies and electrolyte imbalance (101, 102). From the time of conception to birth, human weight increases 6 billion times (103). As a result, it is understandable that fetal metabolic demands must fluctuate considerably during gestation. Similarly, maternal energy expenditure rises during pregnancy, but it is uncertain whether this rise is simply proportional to increased tissue mass and food consumption (104).

Micronutrients (essential vitamins and minerals) are the dietary elements needed in small quantities to sustain nearly all metabolic processes, like cell signaling, motility, proliferation, differentiation, and apoptosis, all of which regulate tissue growth, function, and homeostasis. In early life, these key biological roles allow the fetus to develop and mature into a healthy neonate (105). Due to a chronically inadequate diet, women in low-income countries are frequently unable to achieve the nutritional demands of pregnancy (24). Thiamine being a water-soluble vitamin with a small half-life and limited body stores is more prone to fall below the normal levels in the blood quickly. A regular, ample supply is necessary to keep up with the increased nutritional demands of pregnancy and lactation (11, 106). During pregnancy, physiological factors like hemodilution, increased metabolic demand, increased GFR, renal perfusion and hyperemesis gravidarum predispose women to the deficiency of thiamine. Additionally, in various countries, the high carbohydrate staple diet, consumption of washed milled rice, postpartum dietary restrictions and consumption of thiaminase-containing foods like pickles, tea etc. further increase the demand for thiamine in the body (28, 30, 81).

## 5. Thiamine deficiency in pregnancy and lactation

In LMIC, resource-poor settings, or places with severe acute malnutrition (SAM) inadequate intakes are the primary cause of thiamine deficiency (23, 107, 108). Secondary thiamine deficiency results from increased demand, such as hyperthyroidism, pregnancy, lactation, and fever. It is also linked to decreased absorption, as shown in protracted diarrheas, and impaired utilization, as seen in severe liver disease (35). In developed countries, despite the availability of dietary thiamine, the prevalent use of processed industrial food, alcoholism and bariatric surgery, hyperemesis gravidarum, prolonged intravenous fluids without alternative sources of nutrition, hemodialysis, anorexia nervosa, and magnesium depletion remain the most common causes of thiamine deficiency (39, 109–116). Apart from these, the states of increased thiamine requirements like malignancy (117, 118), fever and infection/sepsis (119), refeeding syndrome (120), and high carbohydrate diets (121) precipitate thiamine deficiency. The increased loss of thiamine during hemodialysis and peritoneal dialysis (122, 123), chronic diuretic therapy (124), and retracted vomiting/diarrhea (125, 126) leads to depletion of the body stores of thiamine. Decreased thiamine intake or absorption in chronic alcoholism (127), bariatric surgery (128), malnutrition (15), restrictive or poor quality diet (129), parenteral nutrition (130), and foods containing thiamine antagonists and thiaminases (39, 131).

A study by Dias et al. conducted among women in a rural, low-income community in Brazil found that thiamine diphosphate levels were inversely correlated with the psychiatric parameters in the Hamilton Anxiety Scale (HAMA) and the Beck Depression Inventory (BDI) tests (132). A retrospective observational study by Hilal Ahmad et al. among peripartum women of Kashmir reported that thiamine-responsive polyneuropathy was present in 27 out of 43 pregnant/puerperium women in Kashmir who presented with weakness and sensory complaints such as numbness or paraesthesia (28). Hegde et al. presented a case series of rare neurological and cardio-pulmonary manifestations of thiamine deficiency in five women during pregnancy and lactation. Three among five had Wernicke’s encephalopathy and two had heart failure. All the women responded to intravenous doses (500 mg) twice for 3 days with regression of all symptoms and were followed by oral supplementation of 100–200 mg/day for a minimum of 6 months (133). Chandwani et al. (134) reported two cases of Wernicke’s encephalopathy following hyperemesis gravidarum. Both the women had MRI-documented WE and received intravenous thiamine; a significant neurological recovery was reported. However, one of the women died later on due to central pontine myelinolysis (134). Whitfield and colleagues reported a high prevalence of thiamine deficiency among a representative sample of Cambodian women of childbearing age (15 ± 49 years) and their young children (6 ± 69 months). Since the cut-off levels for thiamine during pregnancy and lactation have not been defined, the authors used two different cut-off levels for determining the burden of thiamine deficiency in this population. Using the most conservative cut-off of whole blood thiamine levels < 120 nmol/L, 27% of mothers and 15% of children were thiamine deficient, however, prevalence rates of deficiency were as high as 78% for mothers and 58% for children using a cut-off of < 180 nmol/L (135). Whitfield et al. (136) in yet another study assessing the thiamine and riboflavin status among women of childbearing age in rural Prey Veng and urban Phnom Penh, Cambodia has reported a thiamine deficiency (whole-blood thiamine ≤ 90 nmol/L) of 39% among urban and 59% among rural women (*P* < 0.001).

Green et al., conducted a case study of thiamine fortification in Kuria atoll, Republic of Kiribati. A total of 104 households were surveyed and 90 men, 17 pregnant, 44 lactating, and 41 other women of reproductive age were assessed. The prevalence of inadequate thiamine intakes was highest for pregnant women and lowest for men but was > 30% in all groups. Rice was reported to be the optimal vehicle for fortification at a rate of 0.3 mg per 100 g which reduced the prevalence of inadequate intake to 2% or less for all population groups (137). Mary Koshy et al. (138) delineated a study on thiamine deficiency among 24 peripartum women in a rural population in Assam presenting as clinically overt peripheral polyneuropathy. All women received parenteral thiamine (200 mg) daily for 7 days followed by oral thiamine twice a day from the time of discharge until the next review. 90% (18) of patients reported either improvement in neurological deficits or improvement in nerve conduction studies after an average of 10 days (138). Rees and colleagues assessed the nutritional status of women (*n* = 83) in the first trimester of pregnancy in the United Kingdom. They reported that 34% (*n* = 31) had low thiamine status and of these 24 had marginal thiamine status (ETKAC 1.15–1.25) and seven were thiamine deficient (ETKAC > 1.25) (139). In a double-blind randomized clinical trial conducted by Whitfield et al. (140), 90 pregnant women were recruited in the Prey Veng province, Cambodia to determine the maternal thiamine levels at baseline and in breastmilk and among infants at end-line after the consumption of thiamine-fortified fish sauce. The women were randomized into three groups for *ad libitum* fish sauce consumption for 6 months: control (no thiamine), low-concentration (2 g/L), or high-concentration (8 g/L) fish sauce. It was reported that whole-blood thiamine of women in the control group (193 nM; 95% CI, 164–222 nM) was lower than women in the low (282 nM; 95% CI, 164–310 nM; *P* < 0.001) and high groups (254 nM; 95% CI, 225–284 nM; *P* = 0.004). Infants of mothers in the high group (257 nM; 95% CI, 222–291 nM) had higher whole blood thiamine compared with infants of mothers in the control group (187 nM; 95% CI, 155- 218 nM; *P* = 0.004), but not the low group (212 nM; 95% CI 181–244 nM; *P* = 0.07) (140). Another double-blind, 4-parallel arm randomized control trial was conducted by Gallant and colleagues among mothers (2 weeks postpartum) of Kampong Thom, Cambodia to assess the impact of different doses of thiamine supplementation on milk and blood thiamine status. Women (*n* = 335) were randomly assigned to consume one capsule containing 0, 1.2, 2.4, or 10 mg of thiamine daily from 2 to 24 weeks postpartum. It was reported that to reach a milk total thiamine concentration of 191 g/L, a supplemental thiamine dose of 2.35 mg/day was necessary but 1.2 mg/day for 22 weeks was enough to raise milk thiamine concentrations to levels reached by greater supplementation dosages (2.4 and 10 mg/day) and were also comparable to those of healthy mothers in areas free of beriberi (141).

## 6. Clinical consequences of thiamine deficiency in pregnancy and lactation

**FIGURE 1:**
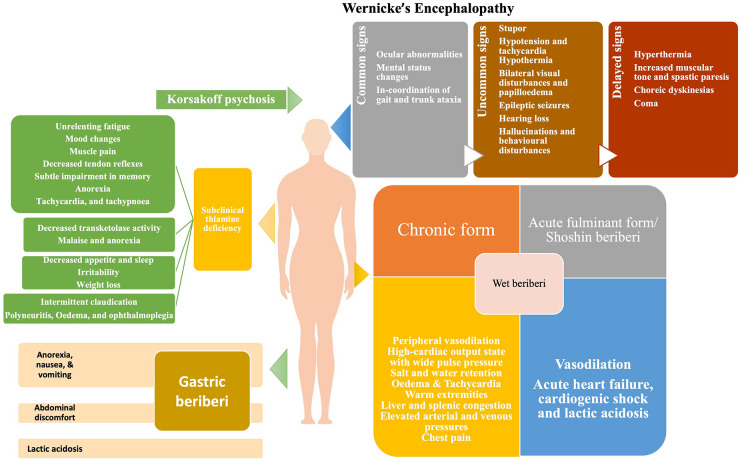
Clinical manifestations of thiamine deficiency.

**FIGURE 2:**
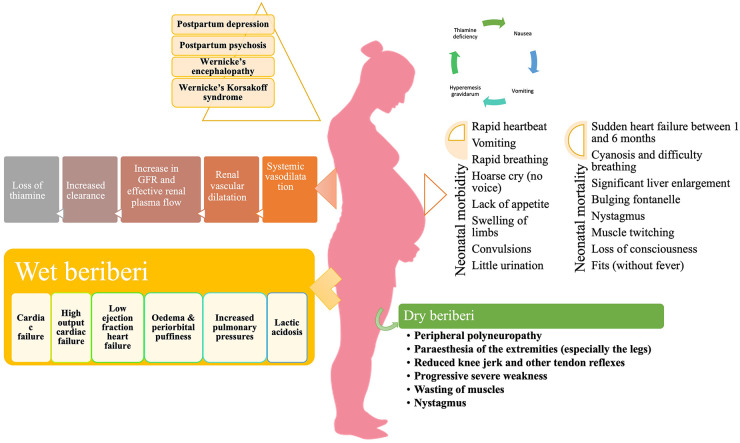
Clinical consequences of thiamine deficiency in pregnancy.

The signs and symptoms of thiamine deficiency, in an individual, vary widely depending upon the clinical setting, the patient’s age and the genetic susceptibility (142). As discussed above, the pathological mechanism of thiamine deficiency includes decreased energy synthesis from mitochondria in the form of ATP when pyruvate-generating substrates (e.g., glucose) are used, as well as increased oxidative stress (143). The deficiency of thiamine can present as a broad range of overlapping disorders ranging from neurological deficits to cardiovascular symptoms as well as gastrointestinal form, (Figure 1) collectively known as thiamine deficiency disorders (TDDs) (81). Colloquially, the term “beriberi” was initially used to describe the spectrum of disorders caused by thiamine deficiency which was further divided into two forms: dry beriberi and wet beriberi depending upon the organ system affected. Wet beriberi primarily affects the cardiovascular system and is associated with edema high cardiac output; ventricular failure; and pulmonary congestion while as dry beriberi predominantly affects the peripheral nervous system and is characterized by polyneuropathy, reduced knee jerk and other tendon reflexes, and progressive severe weakness of muscles. Additionally, other forms of the disease include shoshin beriberi, infantile beriberi, gastric beriberi, Wernicke’s encephalopathy (WE) and Wernicke-Korsakoff syndrome (14). In addition to these manifestations, thiamine deficiency may prove fatal to the developing fetus as well as to the mother (Figure 2). The clinical consequences of thiamine deficiency exclusively during pregnancy and lactation have not been explored fully. However, a number of scattered cases with classical features have been reported from various parts of the world.

### 6.1. Subclinical thiamine deficiency

The initial signs of thiamine deficiency are vague that might easily be linked to a variety of illnesses. The presenting symptoms include unrelenting fatigue, mood changes, muscle pain, decreased tendon reflexes, subtle impairment in memory, anorexia, tachycardia, and tachypnoea (144–146). Various reports suggest that initial symptoms like GI disturbance (nausea, vomiting, discomfort, and dysmotility) may be the primary signs of thiamine deficiency (144, 147). The GI symptoms of thiamine deficiency are often confused with pregnancy-related nausea and vomiting, making the early diagnosis difficult.

### 6.2. Wet beriberi

Cardiomyocytes require a steady supply of energy, and thiamine insufficiency causes disruptions in aerobic respiration pathways, interfering with proper heart function (148). The involvement of the cardiovascular system may take two forms: chronic form or acute fulminant form (Shoshin beriberi). The chronic form comprises of three phases. The first phase is marked by peripheral vasodilation resulting in a high-cardiac output state, wide pulse pressure, tachycardia, sweating, and warm skin. This results in salt and water retention, regulated by the renin-angiotensin-aldosterone system of the kidneys. In the second phase, as the vasodilation progresses further, the kidneys sense a relative reduction of volume and adapt by retaining salt resulting in edema. The third phase is characterized by considerable edema which subjects the heart to a very high workload in order to produce the requisite cardiac output. This causes an overuse injury to cardiomyocytes, resulting in tachycardia, edema, warm extremities, liver and splenic congestion and elevated arterial and venous pressures which are chiefly manifested as chest pain (149). During pregnancy, the most common cardiac manifestations associated with thiamine deficiency include progressive dyspnoea of NYHA Class 2 to 3, generalized edema, and marked right ventricular dilation (133). Additionally, pericardial effusion, intraventricular septum and mild mitral insufficiency have also been reported (150).

The acute fulminant form of wet beriberi is known as “Shoshin beriberi” in which the vasodilation continues causing acute heart failure, cardiogenic shock, and lactic acidosis (142, 151). In severe deficiency, echocardiography shows dilated ventricles (particularly the right ventricle, which is commonly associated with pulmonary hypertension), a D-shaped left ventricle, and a drastically reduced ejection fraction (152). An immediate response to therapy is noted following intravenous infusion of thiamine (153).

### 6.3. Dry beriberi

Since thiamine has a crucial physiological role in energy metabolism, deficiency leads to a decrease in oxidative metabolism. Biochemical derangements disrupt the supply of ATP to neurons, causing oxidative stress, and decreased synthesis of neurotransmitters [e.g., acetylcholine, glutamate, aspartate, and gamma amino butyric acid (GABA)] which produces a toxic neuroexcitatory state (154, 155). Impairment of the pentose phosphate pathway results in decreased nerve conduction velocity because thiamine participates in the maintenance of myelin sheaths (156). The primary symptoms of dry beriberi include lower limb, bilateral and symmetric, paresthesia, calf muscle tenderness, burning of feet and plantar dysesthesia. The absence of ankle jerk reflexes can determine peripheral neuropathy. Persistent deficiency results in the loss of tendon reflexes, atrophy of the calf and thigh muscles, and, lastly, foot drop, and toe drop (35). Dry beriberi in pregnancy can initially present as poor visual acuity, diplopia, and gait ataxia along with bilateral horizontal nystagmus (157).

*Wernicke’s encephalopathy* is an acute neuropsychiatric disorder caused by thiamine deficiency that is frequently coupled with alcohol misuse because of poor diet, carbohydrate-rich diet, inhibition of intestinal ATPase by alcohol, and depletion of magnesium (158). The deficiency of thiamine in WE is considered to cause apoptotic cell death due to N-methyl-D-aspartate (NMDA) toxicity, resulting in neurological symptoms (159). The classical triad of WE includes nystagmus and ophthalmoplegia, changes in mental status, and gait ataxia (160). The mental changes are mostly the result of thalamic or mammillary body involvement and vary from disorientation to mental sluggishness, apathy, poor awareness of the present situation, difficulty concentrating, and, if untreated, coma, and death (161). Classically, WE occur in alcoholics, however, HIV-infected patients, pregnant women suffering from hyperemesis gravidarum, and postoperative bariatric patients are also at risk (162–164). WE in pregnancy and lactation has been reported widely with symptoms varying from mild horizontal nystagmus, dysarthria, and right hemiparesis to more severe neurological manifestations like edematous splenium of the corpus callosum (165) with ataxia being the commonest manifestation (166). A report suggests memory loss as the presenting feature and bilateral lateral rectus palsy as the most common form of ophthalmoparesis in WE (167).

*Korsakoff psychosis* is a severe form of WE characterized by amnesia, confusion, and confabulation with little or no working memory (168). It occurs in patients, who have previously had WE but did not receive prompt and appropriate thiamine replacement therapy (169). Because of their close relationship, these two disorders are frequently referred to as *Wernicke–Korsakoff syndrome* (170). Pregnant women are especially at risk of developing Wernicke–Korsakoff syndrome due to hyperemesis gravidarum (171). Hyperemesis gravidarum remains the main risk factor for the development of any complication associated with pregnancy and lactation (172). *Wernicke–Korsakoff syndrome* typically develops following malnutrition in patients with prolonged self-neglect, alcohol abuse, and other substance abuse (173). Thiamine deficiency occurs in alcoholics because alcohol is calorie-dense yet nutrient-poor. Furthermore, diarrhea and the low capacity of the liver to store vitamins and the impaired conversion of thiamine to the active compound TPP in alcoholics and substance abusers results in impaired storage of thiamine (174). In addition, Korsakoff’s syndrome in alcoholics may be caused by the build-up of lesions during recurrent subclinical episodes of thiamine shortage (175, 176), with ethanol neurotoxicity possibly playing a role (177, 178). Treatment of Wernicke’s encephalopathy with appropriate doses of parenteral thiamine may avoid the development of Korsakoff’s syndrome if administered promptly. According to the guidelines of the Royal College of Physicians, alcoholic individuals at risk for developing WE may be treated with an intravenous infusion of 500 mg of thiamine hydrochloride in 100 mL of normal saline over the course of 30 minutes, thrice daily for 2–3 days. This must be followed by intramuscular dosing of 250 mg of thiamine daily for 3–5 days (173, 179–181).

### 6.4. Gastric beriberi

Donnino (144), reported an unusual, under-recognized form of a gastrointestinal syndrome associated with thiamine deficiency characterized by anorexia, nausea, vomiting, abdominal discomfort, and lactic acidosis, for the first time which came to be known as gastric beriberi (144, 182). However, some consider it to be the prodrome of a more sinister form of thiamine deficiency i.e. WE or that it could be the effect of the thiamine deficient state itself (147). It is well recognized that insufficiency of thiamine causes decreased ATP production in the liver raising the liver enzymes and leading to gastrointestinal symptoms. Our team have also reported various manifestations of gastric beriberi in the otherwise well-fed population of Kashmir (183). Also, vomiting alone has been linked to lower activity of erythrocyte transketolase (33). In addition, to the already known risk factors for beriberi, gastric beriberi may be more common in gastric resection (for malignancy), bariatric surgery, total gastrectomy, Billroth II gastrectomy, or Roux-en-Y diversion (184).

### 6.5. Infantile beriberi

**FIGURE 3:**
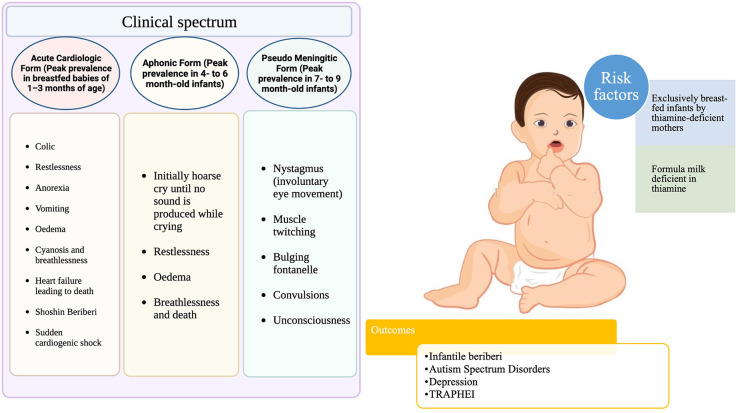
Clinical consequences of thiamine deficiency in infants.

Due to rapid growth, high energy demand, and a high metabolic rate, the thiamine requirements in infancy are high relative to body size. This places newborns at the highest risk of thiamine deficiency in the first year of life (135). In the first months of life, new-borns are more vulnerable to the effects of thiamine deficiency (Figure 3), and exclusively breastfed infants of thiamine-deficient mothers are at the greatest danger (17), which can result in death within hours of clinical presentation if left untreated (26). Infantile beriberi can present as vomiting, refusal to breastfeed, irritability, and constant loud crying which, in some cases, progresses to a “silent cry,” or aphonia (185). If at this stage thiamine is not administered, it can progress into signs and symptoms of congestive heart failure, like tachypnoea, tachycardia, pulmonary edema, hepatomegaly, and, occasionally, cyanosis occurs; at this point, clinical deterioration is generally rapid (108, 186). Many reports suggest that mechanical rice milling coupled with dietary taboos during the postpartum period and formula feeding leads to acute life-threatening metabolic acidosis and pulmonary hypertension in exclusively breastfed infants (187–191). Children who survive thiamine-deficiency-related WE continue to have developmental problems such as impairments in syntax, morphology, reading, phonological working memory, lexical abilities, epilepsy, motor function loss, and intellectual disability (192, 193).

## 7. Genetic defects associated with thiamine deficiency

Mutations in genes encoding for thiamine transporters and metabolizing enzymes cause symptoms similar to those observed in nutrition-based thiamine shortage and correspond with illnesses of mitochondrial malfunction (194). There are four main genetic defects reported: three of which are primarily of neurological phenotype (SLC19A3, SLC25A19, and TPK1) and one with a multisystem disease (SLC19A2) (195, 196). The defects in thiamine transporter genes form the primary cause of poor intestinal thiamine absorption and, consequently, the inadequate cellular distribution of thiamine throughout the body (197).

### 7.1. SLC19A2

SLC19A2 is a thiamine transporter gene found on the membrane of numerous human tissues, including that of the gastrointestinal tract and encoding the hTHTR1, which aids in the transfer of thiamine across the cell membrane (198). Homozygous, heterozygous or missense mutations in SLC19A2 cause an autosomal recessive condition known as thiamine-responsive megaloblastic anemia (TRMA) or thiamine metabolism dysfunction syndrome 1 or Roger’s syndrome (OMIM 249270) (199, 200). This condition primarily occurs in populations with consanguineous partners and is characterized by a clinical core triad of megaloblastic anemia, non-autoimmune diabetes mellitus, and early-onset sensorineural deafness (201). Cardinal features manifest in infancy or adolescence (202, 203). Other manifestations include cardiac abnormalities, optic atrophy, retinal abnormalities, stroke or epileptic-like episodes and short stature (204, 205). High-dose thiamine supplementation has been shown to delay the onset of diabetes, and generate immediate hematopoietic response but does not affect sensorineural hearing loss in children (200, 206, 207).

### 7.2. SLC19A3

Mutations in SLC19A3 (encoding hTHTR2) cause a neurometabolic autosomal recessive disorder known as thiamine metabolism dysfunction syndrome type 2 or biotin-responsive basal ganglia disease (BBGD) or biotin-thiamine responsive basal ganglia disease (BTBGD; OMIM: 607483). This was first described in the Arabian population with a high degree of parental consanguinity and a common missense mutation, p.thr422ala (208). Depending upon the age of onset, this disease is categorized as classical childhood BBGD, early-infantile Leigh-like syndrome/atypical infantile spasms and adult Wernicke’s-like encephalopathy (209). The disease is characterized by acute and recurring episodes of encephalopathy (often triggered by febrile illness, trauma, vaccines), dystonia, rigidity, confusion, difficulties with speech and swallowing, dysarthria, external ophthalmoplegia and seizures, in association with symmetrically distributed brain lesions in the caudate nuclei, putamen, medial thalami and, less frequently, cerebral cortex, brainstem and cerebellum (210–212). Early treatment with biotin (5–10 mg/kg/day) in combination with thiamine (100–900 mg/day) effectively reverses the disease (213, 214).

### 7.3. SLC25A19

SLC25A19 encodes mitochondrial thiamine transporter and so far three distinct missense mutations have been discovered affecting this gene (215). The two mutations, G125S and S194P, collectively cause an autosomal recessive disease known as thiamine metabolism dysfunction syndrome-4 [(THMD4) (Bilateral striatal degeneration and progressive polyneuropathy type) (OMIM 613710)] and is characterized by childhood episodic encephalopathy, often associated with a febrile illness, causing transient neurologic dysfunction and a slowly progressive axonal polyneuropathy (216). A third homozygous mutation, G177A, causes a severe autosomal recessive disorder called Amish lethal microcephaly (MCPHA; OMIM 607196). This disease is characterized by microcephaly apparent at birth, markedly delayed psychomotor development, brain abnormalities, and episodic encephalopathy associated with lactic acidosis and -ketoglutaric aciduria (217).

### 7.4. TPK1

TPK1 converts free thiamine to TPP, as such any mutation in the TPK1 sequence prevents the phosphorylation of thiamine to an active isomer. The onset of the disease is usually early childhood with clinical features of acute encephalopathic episodes, elevated serum, and CSF lactate, learning difficulties, progressive motor dysfunction (e.g., gait abnormalities, ataxia, dystonia, and spasticity) in the striatal, basal ganglia, and cerebellar parts of the brain, while cognition appeared to be unaffected. An early diagnosis is warranted as the condition largely remains treatable, however, diagnosis is delayed due to clinical overlap with other metabolic diseases, including Leigh syndrome (196). The treatment chiefly depends upon oral thiamine supplementation (100–200; 500 mg/day) (201, 218) or thiamine in combination with niacin, biotin, α-lipoic acid, and ketogenic diet (219, 220).

## 8. Prevalence of thiamine deficiency in women and infants

In addition to poor maternal health, thiamine deficiency can be associated with substantial neonatal risks and outcomes. Thiamine deficiency during pregnancy and lactation can present either with subclinical features, maternal polyneuropathy, WE, high-output heart failure, recurrent nausea and vomiting and depressive and anxious symptoms (132) or as infantile beriberi (221).

There are several sporadic cases of thiamine deficiency during pregnancy, lactation (Table 1) and early infancy (Table 2) in many communities especially in Southeast Asia, South Asia and West Africa (34, 187, 190, 222). These regions predominantly fall in LMIC, use polished rice or cassava as primary staples, and are susceptible to food shortages.

**TABLE 1:** The published cases of thiamine deficiency among infants and children.

References	Country	*N*	Population	Clinical presentation	Treatment	Outcome
Bhat et al. (189)	India	29	Infants	Acute onset pulmonary artery hypertension	Intravenous thiamine 100 mg/kg IV followed by 10 mg/day till introduction of supplementary feeds.	Resolution of shock, metabolic complications and pulmonary hypertension
Porter et al. (34)	Cambodia	62 (20 cases and 42 controls)	Infants	Infants with hepatomegaly (liver edge ≥ 2 cm below the costal margin), respiratory rate ≥ 40/min, heart rate ≥ 140/min, and temperature < 37.5°C.	100 mg of thiamine i.m. divided into 3 doses delivered over 90 min. The treatment was repeated at 24 and 48 h after presentation	Thiamine deficiency produces echocardiographic evidence of right ventricular dysfunction, but this evidence is not apparent until deficiency is severe.
Qureshi et al. (190)	India	23	Infants	Acute life-threatening metabolic acidosis due to thiamine deficiency.	Mechanical ventilation, thiamine, saline boluses, dopamine, calcium gluconate, bicarbonate, and antibiotics. Thiamine was given at admission as 100 mg infusion to all infants.	Moaning and irritability subsiding in 2 h and tachycardia in 4 h. Adequate perfusion was achieved in 1 h.
Barennes et al. (32)	Laos	54 infants retrospectively with sudden onset of cardiac failure and 127 prospectively. 127 mothers	Infants and mothers	Acute symptoms in previously healthy breastfeeding infants associated with cardiac failure (tachypnea > 50/min, tachycardia > 170/min, gallop, hepatomegaly > 3 finger’s breadth) or loss of voice.	Thiamine tablets 100 mg, daily for 20 days and infants with suspected thiamine deficiency were treated with thiamine tablets, 30 mg per day for 20 days. Patients with acute symptomatic thiamine deficiency received an intramuscular or slow intravenous injection of thiamine (100 mg i.m. for mothers and 50 mg for infants).	Of the 54 infants with cardiac failure, 49 (90.7%) were cured after thiamine administration Of prospective subjects, all recovered after thiamine administration.
Thankaraj et al. (16)	India	28	Breastfed infants	Short history of vomiting, breathlessness and poor feeding. All infants presented in a critically ill state with prolonged capillary refill time (93%), tachycardia (93%), seizures (36%) and severe respiratory distress (92%).	100 mg of intravenous thiamine bolus within 1 h of admission, followed by 100 mg intravenously for a minimum of 7 days till discharge. All infants received other treatment modalities as per the pediatric protocol for treatment of shock in the hospital.	Dramatic clinical resolution of shock within 24 h of receiving intravenous thiamine (100 mg) bolus.
Fattal-Valevski et al. (252)	Israel	9	Infants	Vomiting, lethargy, irritability, abdominal distension, diarrhea, respiratory symptoms, developmental delay, and failure to thrive.	Intramuscular thiamine 50 mg/day for 14 days and a supportive treatment which was determined by the attending physician.	Clinical signs and symptoms disappeared completely within 2 to 3 weeks of treatment with very rapid normalization of TPPE, occurring 1 to 7 days after treatment.
Narasimha Rao et al. (270)	India	166	Children	Altered sensorium, acute loss of milestones, ptosis, irregular respiration, with a raised serum and CSF lactate. External ophthalmoplegia, seizures, hypotonia, aphonia, choreo-athetoid movements, and arreflexia	Thiamine supplementation (200–300 mg per day) followed by 75 mg daily for 3 months after discharge	Within 24 h the first signs of improvement in the level of consciousness, respiratory abnormalities and ptosis occurred whereas head control, tone, involuntary movements and milestones recovered partially over next few weeks. Developmental delay and hypotonia remained at 3–6 months follow-up in two cases with persistent computed tomography head lesions
Coats et al. (229)	Cambodia	27	Infants	Hepatomegaly (liver edge > 2 cm below the right costal margin), respiratory rate > 40, heart rate > 140, absence of fever, and at least 2 of the following: aphonia or dysphonia; wheezing on exam; decreased urine output; recent vomiting or spitting up; and increased irritability.	3 doses of thiamine (25, 25, then 50 mg) administered 30 min apart and repeated on the subsequent 2 days	The respiratory rate decreased by ≥ 10 breaths per minute in 26% by 24 h and in 38% by 72 h. Heart rate decreased by ≥ 20 beats per minute in 30% by 24 h and in 33% by 72 h. The mean baseline TDP blood level of infants with and without objective findings of improvement at 24 h was 48 nmol/L (95% CI: 35, 61) and 48 nmol/L (95% CI: 36, 60) (*P* = 0.82).
Rao and Chandak (271)	India	55	Infants	Tachypnoea, chest in drawing, tachycardia, aphonia, external ophthalmoplegia and cardiomegaly with dilatation of the right heart and pulmonary hypertension on 2D-echocardiography.	75 mg of intramuscular thiamine twice a day for 5 days in addition to routine supportive care.	Reversal of echocardiographic abnormalities in 19’s at 2–3 week follow-up
Wani et al. (272)	India	58	Infants	Infantile encephalitic beriberi: altered state of consciousness, seizures, and altered personality or cognition	100-mg intravenous infusion of thiamine	Regression of basal ganglia hyper-echogenicity after thiamine administration, with almost normal appearance of basal ganglia in 2–4 weeks in 18 infants and complete resolution of basal ganglia hyper-echogenicity at 4–8 weeks, in 8 infants.
Sastry et al. (188)	India	231	Infants	Fast breathing, poor feeding, vomiting, aphonia, tachypnoea, tachycardia and hepatomegaly. Echocardiogram revealed a grossly dilated right heart with severe pulmonary hypertension	Intravenous thiamine 100 mg infusion diluted in 10 mL of normal saline over 1 h, once a day for 3 days	Complete resolution of pulmonary hypertension in 92% of cases within 24–48 h Acceptance of feeds and cessation of vomiting within 6 h. Hepatomegaly, tachypnoea and tachycardia reduced within 24 h.

**TABLE 2:** The studies reporting thiamine deficiency among women.

References	Country	*N*	Population	Clinical presentation	Treatment	Outcome
McGready et al. (29)	Karen	25	Pregnant women	Peripheral numbness, cramping or aching muscles, and anorexia,	Oral thiamine hydrochloride (100 mg/day) until delivery	Peripheral numbness and tingling and cramping or aching muscles appeared to resolve with thiamine supplementation.
Mary Koshy et al. (138)	India	24	Peripartum women	Muscle weakness of upper and/lower limbs less than Grade 5, positive sensory symptoms, Objective sensory deficits, absent or reduced deep tendon reflexes and an abnormal nerve conduction study	Parenteral (intramuscular or intravenous) thiamine 200 mg per day for an average of 7 days, followed by 33 mg thiamine orally twice a day at discharge until the next follow-up	90% of cases reported either improvement of neurological deficits or improvement in nerve conduction studies after an average of 10 days.
Hilal Ahmad et al. (28)	India	29	Peripartum women	Peripheral neuropathy with generalized weakness and or sensory symptoms in the form of paraesthesia’s or numbness in limbs	200–500 mg Intravenous thiamine thrice a day for 3–5 days, followed by oral thiamine.	27 patients showed resolution of edema, improvement in weakness, altered mental status, ophthalmoparesis, and nystagmus within 24–72 h.
Divya et al. (245)	India	6	Pregnant women	Hyperemesis gravidarum with neurological abnormalities like slurred speech, visual loss, seizure and aggressive behavior in all woman while one woman had typical clinical triad of Wernicke’s encephalopathy	Parenteral thiamine in all followed by 100 mg orally for 2 months in three patients	Atypical neurological signs and symptoms following hyperemesis gravidarum invariably responded immediately to appropriate dosage of parenteral thiamine.
Meggs et al. (273)	USA	1	Pregnant women	Nausea, emesis, right upper quadrant abdominal pain, altered mental status, internuclear ophthalmoplegia with bilateral nystagmus.	Intravenous thiamine	Not reported
Indraccolo et al. (150)	USA	1	Pregnant women	Hyperemesis gravidarum, mental confusion, horizontal nystagmus, aphasia, ataxia. Wernicke’s encephalopathy	300 mg I.V. thiamine in three 100-mg doses	Remission of fever, nystagmus, improvement in hemodynamic parameters and recovery from coma.

### 8.1. Gambia

Most of the reports of thiamine deficiency in Sub-Saharan Africa have been occasional, involving groups of refugees, soldiers, prisoners, and a few distant communities (223–227). A recent study by Bourassa and colleagues assessed the thiamine status of women of reproductive age living in rural Gambia and the association between thiamine status and seasonality. The study reported that around 35.8% of the sample was at high risk of thiamine deficiency (ETKac ≥ 1.25) and the risk of thiamine deficiency was significantly higher in the wet (47.9%) compared with the dry season (22.9%) (*P* < 0.001) (30). The reliance on rice remains an important risk factor among Gambian people, especially women, and the cases peak in the rainy season when food supplies are lowest and there is intense agricultural activity with increased energy expenditure (228).

### 8.2. Cambodia

Thiamine deficiency disorders and suboptimal thiamine status have recently been identified in Cambodia (135). A case-control study conducted by Coats et al. evaluated the whole blood ThDP levels in 27 infants diagnosed with beriberi and their mothers and matched them with healthy Cambodian and American controls. Surprisingly, there was no significant difference in whole blood ThDP levels between Cambodian mothers and infants with and without beriberi but both pairs of Cambodian women and infants had much lower ThDP levels than the American controls (229). Another cross-sectional study was conducted by Whitfield et al. to assess the status of thiamine in women of childbearing age in rural and urban Cambodia, compared with women in Canada. The study reported that thiamine deficiency (ThDP ≤ 90 nmol/L) was common among both urban (39%) and rural (59%) Cambodian women (*P* < 0.001), whereas <20% of Vancouver women were thiamine deficient (*P* < 0.001) (136). A similar study by Whitfield et al. among a nationally representative sample of Cambodian women of childbearing age (15 ± 49 years) and their young children (6 ± 69 months) found that women had a lower mean (95% CI) ThDP of 150 nmol/L (146 ± 153) compared to children, 174 nmol/L (171 ± 179; *P* < 0.001). Using different cut-off values for ThDP, 27–78% of women and 15–58% of children were thiamine deficient, which was especially prevalent among infants aged 6 ± 12 months (135). In addition, various studies have reported cases of infantile beriberi from different areas of Cambodia (34, 186, 230).

### 8.3. Thailand

A study by McGready et al. (29) among the postpartum Karen refugee population has reported that at 3 months postpartum, thiamine deficiency, reflected by ETKA ≥ 1.20% was found in 57.7% of mothers, 26.9% of whom had severe deficiency (ETKA > 1.25%). A number of studies report childhood thiamine deficiency and the high infant mortality attributed to the deficiency of thiamine (26, 231, 232). A study by Stuetz et al. evaluated the ThDP in whole blood and thiamine in the breast milk of lactating women. It was found that the prevalence of women with low whole blood ThDP (<65 nmol/L) was 5% and with deficient breast-milk total thiamine (<300 nmol/L) was 4% (233). Further, thiamine deficiency was also reported among children with malaria, parasitic infection and acute diarrhea (27, 234, 235). A recent study reported an outbreak of peripheral neuropathy in Bueng Kan Provincial Prison in northeast Thailand associated with thiamine deficiency (236).

### 8.4. India

Thiamine deficiency has primarily been reported from Northeast India, especially Kashmir, with some scattered cases from South India (221, 237). A number of reports of infantile beriberi have been reported from North India primarily due to postpartum customary dietary restrictions (16, 238–240). A number of case reports have documented WE in women with hyperemesis gravidarum which underlines the significance of thiamine supplementation for pregnant women experiencing protracted vomiting (134, 166, 241–245). Thiamine-responsive acute pulmonary hypertension of early infancy and acute life-threatening metabolic acidosis in exclusively breast-fed infants has been reported intermittently from North India, especially Kashmir (188–190, 246). Another recent study from Kashmir has reported thiamine-responsive neuropathy among peripartum women (28). A case series by Hegde et al. (133) reported the neurological and cardio-pulmonary manifestations of thiamine deficiency in pregnancy and lactation. A case report from the Siaha district of Mizoram, India bordering Myanmar reported that mothers taking the kuhva (raw areca nut) daily during the last pregnancy were independently associated with higher infant mortality (247).

### 8.5. Laos

Several case series of infantile beriberi and suboptimal thiamine status among children have been reported from Laos. The assessment of a cohort of 778 sick infants (0–12 months) found that 13.4% have thiamine deficiency and are defined as basal ETK activity < 0.59 (33). A large-scale cross-sectional survey of 22 villages in Luang Namtha Province found that of 468 live-born infants, 50 (10.6%, 95% CI: 8.0–13.8) died during the first year. According to verbal autopsies, 17 deaths (3.6%) were caused by suspected infantile thiamine deficiency. Of 127 mothers, 60 (47.2%) reported edema and paraesthesia; while among 127 infants, 2 (1.6%) had probable thiamine deficiency, and 8 (6.8%) had possible thiamine deficiency (32).

### 8.6. Myanmar

Thiamine deficiency has been reported among infants from resource-poor settings of Myanmar, like refugee camps, and displaced communities (248). It is the second most common cause of infant deaths between 28 days and 1 year of age in Myanmar (249). A nationally representative survey of infant mortality reported that 0.3% of live births died from TDDs before 5 years of age (23, 250).

### 8.7. Other communities

In 2005, several infants fed on thiamine-deficient soy infant formula were admitted to several intensive care units in Israel with encephalopathy and were later diagnosed as suffering from thiamine deficiency (251, 252). Pediatric thiamine deficiency was perceived as being eradicated or anecdotal in high-income countries, however, from 2000–2020, 389 cases of pediatric thiamine deficiency have been documented with various predisposing factors including, genetic causes, lifestyle (diabetes, obesity, and excessive consumption of sweetened beverages), eating disorders, cancer, gastrointestinal disorders/surgeries, critical illness, and artificial nutrition (253). A case report of a 10-week-old girl with infantile beriberi from Reunion Island, a French Island, has recently been documented (254).

## 9. Improvement of thiamine status

Diversification of food is the most sustainable approach to prevent micronutrient deficiencies, especially thiamine. Dietary modification, including proper food preparation techniques and reduction in the consumption of anti-thiamine factors, are also known to decrease the loss of thiamine. Lastly, large-scale food fortification programmes and supplementation are the essential strategies for boosting thiamine consumption in areas where clinical and subclinical deficits are common.

### 9.1. Diversification of food

Incorporation of a wide range of thiamine-rich foods like legumes (pulses, beans and groundnuts), lentils etc. in the food basket is a very resourceful approach in improving the thiamine status of the deficient population. Nutrition education along with diversification of food should be encouraged by making vegetable gardening a regular component of all disaster-affected population relief programmes. In places where white-milled rice is a staple food, parboiled undermilled rice should be promoted as a thiamine-rich alternative (255, 256).

### 9.2. Dietary modification

Changing dietary patterns to promote the consumption of thiamine-rich foods while decreasing the consumption of known thiamine antagonists and thiamine-poor foods may improve thiamine intake. The two major approaches to dietary modification include the reduction of losses of thiamine during the preparation and cooking of the meal and the reduction of the intake of anti-thiamine factors. The loss of thiamine during preparation is minimized by reducing the number of washes of the rice before cooking and cooking rice in two volumes of water only (257, 258). Cooking losses of thiamine in vegetables can be decreased by washing vegetables before chopping them into small pieces, keeping cooking time to a minimum, consuming freshly prepared meals immediately, and cooking vegetables in a small amount of water and consuming the water (259).

It is well-documented that anti-thiamine factors including, thiamine antagonists and thiaminases contribute to the development of TDDs, especially in populations with marginal thiamine intake (260). Nutrition education and awareness programmes help ameliorate thiamine deficiency by lowering the intake of anti-thiamine components and improving thiamine nutriture.

### 9.3. Fortification

Food fortification is defined as the practice of deliberately increasing the content of essential micronutrients in food to improve the nutritional quality of the food supply and to provide a public health benefit with minimal risk to health (261). Food fortification is a low-cost, tenable technique for preventing micronutrient deficits (137). Large-scale food fortification of centrally processed food vehicles has a long history of use in industrialized countries for the successful reduction of micronutrient deficiencies and improvement of health outcomes, such as reduced odds for anemia, cretinism, or neural tube defects by providing iron, iodine, or folic acid, respectively (262, 263). Fortification aims to raise the target population’s thiamine consumption to a level consistent with minimal risk of TDDs. Primarily, two thiamine salts are used for fortification: thiamine hydrochloride for liquid vehicles and thiamine mononitrate for dry preparations, however, both are heat labile and destabilized by humidity, light, sulfites and oxygen (264). The vehicle for thiamine fortification must be consumed by the general population throughout the year, must be cost-effective and stable, and should primarily be processed at a centralized facility (265). The most common vehicles for thiamine fortification include rice, wheat flour, corn meal, fish and soy sauces, and salt (81) while sugar, vegetable oil, bouillon cubes, and curry powders are less commonly used vehicles (266). In addition, research is ongoing on biofortification which involves using traditional plant breeding methods, agronomic approaches, or genetic engineering to improve a plant’s micronutrient content (267, 268).

### 9.4. Supplementation

Supplementation refers to the administration of relatively substantial amounts of micronutrients, typically in the form of pills, capsules, or syrups. It has the benefit of being able to provide an appropriate amount of a particular nutrient in a highly absorbable form, and it is frequently the quickest option to control deficiency in people or population groups considered deficient (261). In emergencies, including famines, embargos, refugee settings, etc., thiamine supplementation is the mainstay for averting worse clinical outcomes of thiamine deficiency. Typically, procurement and purchase of micronutrients in somewhat costly pre-packaged form is cost-ineffective and requires high patient compliance. However, as a preventive measure, thiamine tablets can be supplied to pregnant and lactating mothers through prenatal and postnatal clinics to prevent infantile beriberi, especially in exclusively breastfed infants (269).

## 10. Knowledge gaps

There is strong evidence of the occurrence of thiamine deficiency disorders in vulnerable situations like pregnancy and lactation, especially from LMICs. It is surprising that even after the abundant reports on worse health outcomes due to TDDs across various populations, several gaps in knowledge exist. The lack of a holistic case definition of TDDs, incomplete understanding of biomarkers, and the paucity of data on the optimal dose of thiamine supplementation are the major existing limitations. Also, comprehensive data is needed to understand the factors triggering the clinical sequelae, and to determine the relevant cut-offs for thiamine status. Currently, no extensive studies are exploring the genetic aspect of TDDs among different population groups, as genetic variants may enhance the likelihood of severe effects of low thiamine status. The insufficiency of data on at-risk maternal populations across the world limits our understanding of the burden of TDDs and their health implications in terms of neonatal mortality and morbidity.

## 11. Conclusion

Thiamine deficiency during pregnancy and lactation is a common condition reported from LMICs. Maternal physiological changes like increased metabolic demand, renal perfusion, and hyperemesis gravidarum, in conjunction with poor dietary habits and postpartum dietary restrictions, have been the common implicating factors. Despite the substantial advances in scientific understanding of TDDs, many women across the world continue to suffer from the ill effects of insufficient thiamine, particularly in resource-poor areas of Southeast Asia. The considerable maternal complications in terms of WE, high-output heart failure, pulmonary hypertension, postpartum depression, and polyneuropathy can be averted by providing optimum thiamine supplementation. Thiamine deficiency in infants, in the form of infantile beriberi, remains an important cause of neonatal mortality and long-term morbidity in underdeveloped nations. In addition to causing infant mortality, subclinical thiamine deficiency have an unprecedented long-term influence on neurological development in children. Given the vast spectrum of clinical manifestations, the clinical diagnosis remains extremely challenging and deficiency is frequently missed or misdiagnosed in the mother as well as the infant. Thus, raising awareness and sensitizing health and medical professionals regarding the low dietary staples, food practices and clinical features of thiamine deficiency is of paramount importance. Also, population-level thiamine assessments are required to steer supplementation or food fortification initiatives to at-risk populations and subgroups.

## Author contributions

OK and SN conceived the idea. MT critically reviewed the manuscript and edited the manuscript. UM revised the manuscript. OK wrote the manuscript. GB supervised the review and helped in editing. All authors contributed to the article and approved the final version.
